# A phase I/II study of GLIF combination chemotherapy for taxane/platinum-refractory/resistant endometrial cancer (GOGO-EM2)

**DOI:** 10.1007/s00280-018-3648-y

**Published:** 2018-07-20

**Authors:** Yusuke Tanaka, Yutaka Ueda, Satoshi Nakagawa, Shinya Matsuzaki, Eiji Kobayashi, Yasuhiko Shiki, Yukihiro Nishio, Masahiko Takemura, Toshiya Yamamoto, Kenjiro Sawada, Takuji Tomimatsu, Kiyoshi Yoshino, Tadashi Kimura

**Affiliations:** 1 Department of Obstetrics and Gynecology, Graduate School of Medicine, Osaka University, 2-2 Yamadaoka, Suita, Osaka 565-0871 Japan; 2 Osaka Rosai Hospital, 1179-3 Kita-ku, Nagasone-cho, Sakai, Osaka 591-8025 Japan; 3 Osaka Police Hospital, 10-31 Kitayama-cho, Tennoji-ku, Osaka, 543-0035 Japan; 4 Osaka General Medical Center, 3-1-56 Mandai-Higashi, Sumiyoshi-ku, Osaka, 558-8558 Japan; 5 Sakai City Medical Center, 1-1-1 Ebaraji-cho, Nishi-ku, Sakai, Osaka 593-8304 Japan; 6 University of Occupational and Environmental Health, 1-1, Iseigaoka, Yahatanishi-ku, Kitakyushu-shi, Fukuoka, 807-0804 Japan

**Keywords:** Endometrial cancer, Taxane/platinum, Refractory/resistant, GLIF, Combination chemotherapy

## Abstract

**Purpose:**

Development of new treatment strategies for endometrial cancer that has become refractory or resistant to taxane/platinum is a critical need. The present study was a phase I/II study of gemcitabine, levofolinate, irinotecan, and 5-fluorouracil (5-FU) (GLIF) combination chemotherapy to determine optimal dosages, safety, and efficacy.

**Methods:**

Taxane/platinum-resistant or -refractory endometrial disease was defined as tumor progression within 6 months after a taxane/platinum-based regimen. Maximum tolerated dose was investigated by a 3 + 3-designed phase I study. The phase II study was conducted using the recommended doses determined in the phase I study.

**Results:**

The dosages recommended for the phase II trial were determined, in the phase I trial, to be: gemcitabine 800 mg/m^2^, levofolinate 100 mg/m^2^, irinotecan 80 mg/m^2^, and 5-FU 1000 mg/m^2^. Thirty patients were enrolled, including the three patients who received GLIF therapy at the same dose as the recommended phase II dose in the phase I study. Two patients were excluded at this point due to study protocol violations, and the remaining 28 patients were included for analysis. Phase II revealed that the response and disease control rates were 7.1% (2/28) and 39.3% (11/28), respectively, and that the median PFS and OS were 3 months [95% confidence interval (CI) 3–7] and 12 months (95% CI 9–17), respectively. Febrile or grade 4 neutropenia was observed in 14% (4/28) of the cases. Grade 3 or 4 thrombocytopenia was not observed.

**Conclusion:**

We found that GLIF combination chemotherapy is potentially a useful treatment option for endometrial cancers refractory or resistant to taxane/platinum-based chemotherapy.

## Introduction

The incidence of endometrial carcinoma has been increasing. In most patients the tumor is still confined to the uterus at first diagnosis, and there is therefore a good prognosis. However, the prognosis for advanced or recurrent endometrial carcinoma becomes extremely poor [1]. In particular, recurrent endometrial cancers with the shortest treatment-free intervals (TFIs) are associated with the poorest prognoses.

A combination chemotherapy using cisplatin/doxorubicin (AP) has long been the standard regimen for advanced or recurrent endometrial cancer [2]. Recently, a more effective combination chemotherapy, using taxane and platinum, has begun replacing AP therapy as a community standard [3]. Taxane/platinum has been used as a standard first-line regimen for fresh cases, and as a second-line regimen for those previously treated by AP therapy. However, a second-line regimen for cases previously treated with taxane/platinum, with or without anthracycline, is yet to be established. Especially for taxane/platinum-treatment cases with a TFI of 6 months or shorter, development of new treatment strategies has become a critical need.

Previous studies have shown that irinotecan and gemcitabine exhibit good response against advanced or recurrent endometrial cancer cases, respectively, although those cases were not yet resistant to taxane or platinum [4–7]. In a series of consecutively treated patients with metastatic pancreatic cancer, a retrospective study demonstrated the efficacy and safety of a regimen known as G-FLIP, using gemcitabine, 5-FU, leucovorin, irinotecan, and cisplatin as a second-line chemotherapy [8–11]. In the present study, we have investigated the safety and efficacy of a new GLIF combination chemotherapy (using gemcitabine, levofolinate, irinotecan, and 5-FU) against cases with taxane/platinum-resistant or -refractory endometrial cancer. This regimen was modified from the previous G-FLIP regimen by substituting levofolinate for leucovorin and omitting cisplatin.

## Materials and methods

This GOGO-EM2 study, approved by the institutional review board of Osaka University Hospital, was conducted from July 2011 to February 2016. Written informed consent was obtained from all patients.

## Phase I study

### Eligibility criteria

Patients with taxane/platinum-refractory or -resistant endometrial cancer were eligible for the study. Other eligibility criteria included: age ≥ 20 years, Eastern Cooperative Oncology Group performance status of 0–2, measurable or evaluable disease, a life expectancy ≥ 3 months, and adequate hematological, renal, and hepatic function. Taxane/platinum-resistant or -refractory disease was defined as progression during or within 6 months after a taxane/platinum-based regimen.

### Exclusion criteria

The exclusion criteria included: patient with a *28 homozygous or *6 homozygous UGT1A1 gene polymorphism, hypersensitivity to chemotherapeutic drugs, interstitial pneumonia, ascites or pleural effusion that required drainage, active clinically significant inflammatory disease, duplicated cancer, symptomatic brain metastasis, uncontrolled diabetes mellitus, diarrhea, ileus, cardiac disease, edema, or patients whose circumstances did not permit them to complete the study.

### Dose escalation protocol

**Fig. 1:**
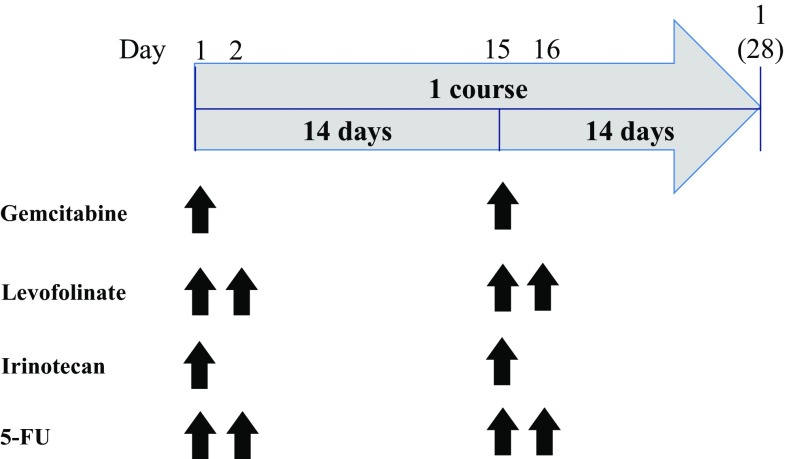
Treatment schedule. Fixed doses of levofolinate (100 mg/m^2^) and 5-FU (1000 mg/m^2^) were administered by intravenous infusion over the course of 90 min on day 1. For the phase I study, at the starting dose level (Level 0), patients received intravenous gemcitabine (800 mg/m^2^ body surface area) over the course of 30 min, and irinotecan (80 mg/m^2^), over the course of 90 min, on days 1 and 15, respectively. Levofolinate (100 mg/m^2^ over 120 min) was administered intravenously on days 1, 2, 15, and 16. For 5-FU, an intravenous bolus 400 mg/m^2^, followed by continuous 600 mg/m^2^ over the course of 480 min, was administered on days 1, 2, 15, and 16, respectively. This schedule was repeated every 4 weeks. The first and second dose escalation indicated 800–1000 and 1200 mg/m^2^, respectively, for gemcitabine and 80–100 and 120 mg/m^2^, respectively, for irinotecan. The first and second dose reduction indicated 800–600 and 500 mg/m^2^, respectively, for gemcitabine and 80–60 and 50 mg/m^2^, respectively, for irinotecan

The treatment schedule we used is shown in Fig. 1. Adverse treatment effects were graded based on WHO criteria for toxicity. For our phase I study of GLIF therapy, the maximum tolerated dose (MTD) levels of gemcitabine and irinotecan were evaluated. At the starting dose level (Level 0), patients received intravenous gemcitabine (800 mg/m^2^ body surface area) over the course of 30 min, and irinotecan (80 mg/m^2^) over the course of 90 min, on days 1 and 15, respectively. Levofolinate (100 mg/m^2^ over 120 min) was administered intravenously on days 1, 2, 15, and 16. For 5-FU, an intravenous bolus of 400 mg/m^2^, followed by continuous infusion of 600 mg/m^2^ over the course of 480 min, was administered on days 1, 2, 15, and 16, respectively. This schedule was repeated every 4 weeks. Granulocyte colony-stimulating factor (G-CSF) was used to support hematopoiesis when grade 4 neutropenia, or grade 3 neutropenia with fever, was observed. A 3 + 3 study design was used for dose escalation. The first and second dose escalation indicated 800–1000 mg/m^2^ and 1200 mg/m^2^, respectively, for gemcitabine and 80–100 mg/m^2^ and 120 mg/m^2^, respectively, for irinotecan. The first and second dose reduction indicated 800–600 mg/m^2^ and 500 mg/m^2^, respectively, for gemcitabine, and 80–60 mg/m^2^ and 50 mg/m^2^, respectively, for irinotecan.

### Determination of MTD

Toxicity was assessed after every treatment cycle, graded according to the Common Terminology Criteria for Adverse Events (CTCAE), version 4.0 [12]. Dose limiting toxicity (DLT) was defined as when grade 4 thrombocytopenia, grade 3 or 4 febrile neutropenia, or grade 3 or 4 non-hematologic toxicity occurred, or when the patient’s condition, even 14 days after the scheduled day of the subsequent cycle, did not meet the starting criteria, as follows: (i) the pretreatment neutrophil count was < 1500 cells/mm^3^, (ii) the pretreatment platelet count was < 75,000/mm^3^, (iii) patients had diarrhea, of any grade, 24 h prior to chemotherapy, (iv) patients had fever ≥ 38 °C, (v) AST or ALT > 2.5 × the upper limit of normal (ULN) was observed, (vi) serum bilirubin > 1.5 × ULN was observed, (vii) pre-treatment serum creatinine level was elevated, or (viii) grade 3/4 non-hematologic toxicity occurred.

For the 3 + 3 design, the first three patients are treated by the starting dose level (Level 0). If no DLT occurs, the dose is escalated for the next cohort of three patients. If one DLT occurs, three additional patients are treated at the same level with dose escalation only if no additional DLT occurs. If two or three DLTs occur, the prior dose level is defined as MTD and recommended as the phase II dose.

## Phase II study

Eligibility and exclusion criteria were the same as in the phase I study. Any and all patients who received GLIF therapy at the same dose as the standard phase II dose in the phase I study were included in the analysis for the phase II study.

### Chemotherapy protocol

Patients received intravenous gemcitabine (800 mg/m^2^ body surface area) over the course of 30 min and irinotecan (80 mg/m^2^) over the course of 90 min on days 1 and 15, respectively. Levofolinate (100 mg/m^2^) over 120 min was administered intravenously on days 1, 2, 15, and 16. For 5-FU, an intravenous bolus of 400 mg/m^2^, followed by continuous 600 mg/m^2^ over the course of 480 min, was administered on days 1, 2, 15, and 16, respectively. This schedule was repeated every 4 weeks, until disease progression was confirmed by diagnostic imaging.

### Toxicity assessment

Toxicity was assessed every treatment cycle and graded according to CTCAE. Subsequent cycles were delayed up to 2 weeks if (i) the pretreatment neutrophil count was < 1500 cells/mm^3^, (ii) the pretreatment platelet count was < 75,000/mm^3^, (iii) patients had diarrhea of any grade 24 h prior to chemotherapy, (iv) patients had fever ≥ 38 °C, (v) AST or ALT > 2.5 × ULN was observed, (vi) serum bilirubin > 1.5 × ULN was observed, (vii) pre-treatment serum creatinine level was elevated, or (viii) grade 3/4 non-hematologic toxicity was observed. Patients who failed to recover adequate counts within a 2-week delay were to discontinue cytotoxic chemotherapy.

Dose adjustments were performed for hematological and other adverse events. A 20% dose reduction of gemcitabine, irinotecan, and 5-FU was done for grade 4 hematologic toxicity in the previous cycle, for diarrhea (grade 2 or worse), or for grade 3 or other non-hematologic toxicity. If these adverse events repeatedly occurred after level 1 dose reduction, another 20% dose reduction was done. The patient had to go off protocol if the following events occurred: (i) disease progression confirmed by a diagnostic imaging, (ii) hematologic/non-hematologic toxicity that violated drug administration criteria—despite a level 2 dose reduction, (iii) allergic reaction (grade 3 or worse), (iv) interstitial pneumonia, (v) hematologic/non-hematologic toxicity failed to recover within a 2-week delay, (vi) grade 4 non-hematologic toxicity, or (vii) patient’s voluntary withdrawal of participation in this study.

### Assessment of chemotherapeutic response

The tumor response to treatment was evaluated by CT scan images after three chemotherapy cycles, according to RECIST guidelines, version1.1. Complete response (CR) was defined as disappearance of all target lesions, and partial response (PR) as at least a 30% decrease in the sum of diameters of target lesions, taking as reference the baseline sum diameters. Progressive disease (PD) was defined as at least a 20% increase in the sum of diameters of target lesions or the appearance of a new lesion, and stable disease (SD) as neither sufficient shrinkage to qualify for PR nor sufficient increase to qualify for PD. Those cases that had confirmed new lesions before completion of three cycles of chemotherapy were also defined as PD.

### Outcome measurements

Primary outcomes were response rate and toxicity. Secondary outcomes included progression-free survival (PFS) and overall survival (OS). PFS was defined as the interval from registration to the date of diseases progression—confirmed by diagnostic imaging. OS was defined as the interval from registration to the last follow-up or death. The median PFS and OS were estimated using the Kaplan–Meier method. Statistical analysis was conducted using JMP Pro 13 statistical software (SAS Institute Inc., Cary, NC, USA).

## Results

### Phase I study

During the phase I component of this study, assessable patients were enrolled to receive each dose level (Table 1). Initially, three patients were tested at the starting doses of 800 mg/m^2^ for gemcitabine and 80 mg/m^2^ for irinotecan (Level 0). No patient encountered a DLT at these dose levels; therefore, the doses were escalated to 1000 mg/m^2^ for gemcitabine and 100 mg/m^2^ for irinotecan, respectively (Level 1). Two of the three patients encountered DLTs. Thus, Level 0 was considered to be the MTD. We proceeded with this as the recommended phase II dosage.

**Table 1:** Phase I patient characteristics and study results

Phase I study	Case 1	Case 2	Case 3	Case 4	Case 5	Case 6
Dose escalation level^a^	0	0	0	1	1	1
Age (years)	62	71	61	56	60	54
Disease status	Advanced	Recurrent	Advanced	Advanced	Recurrent	Advanced
Histology	G2 endometrioid	G3 endometrioid	Serous	G2 endometrioid	G2 endometrioid	G3 endometrioid
Previous radiotherapy	No	No	No	No	No	No
Taxane/platinum regimen	TC	TC	TC	TEC	TEC	TC
Response to taxane/platinum regimen	Refractory	Resistant	Refractory	Refractory	Resistant	Refractory
DLT	No	No	No	Febrile neutropenia	No	Febrile neutropenia

^a^Dose escalation level	Gemcitabine (mg/m^2^)	Levofolinate (mg/m^2^)	Irinotecan (mg/m^2^)	5-FU (mg/m^2^)
Level − 2	500	100	50	1000
Level − 1	600	100	60	1000
Level 0	800	100	80	1000
Level 1	1000	100	100	1000
Level 2	1200	100	120	1000

## Phase II study

### Patients’ characteristics

From November 2011 to July 2015, 30 patients were enrolled, including the 3 patients who received GLIF therapy at the same dose as the recommended phase II dose in the phase I study. Two patients were excluded at this point due to study protocol violations, and the remaining 28 patients were included for analysis. The characteristics of these 28 patients are summarized in Table 2. The median age was 65 years (range 33–76). Staging was undertaken according to the 2009 International Federation of Gynecology and Obstetrics (FIGO) staging system. Seventeen of the 28 patients had advanced-stage diseases (stage III or IV). All patients were histopathologically diagnosed, and the histological subtype included endometrioid carcinoma in 17 patients, serous carcinoma in 4, clear cell carcinoma in 3, poorly differentiated carcinoma in 1, mixed carcinoma in 1, and carcinosarcoma in 2, respectively. Four of the 28 patients had previously received radiotherapy before registering for this study.

**Table 2:** Phase II patient characteristics

Total number of patients	28
Median age (range, years)	65 (33–76)
Disease status	
Advanced	5
Recurrent	23
Histology	
Endometrioid	
G1	1
G2	8
G3	8
Serous	4
Clear cell	3
Poorly differentiated	1
Mixed	1
Carcinosarcoma	2
Previous radiotherapy	
Yes	4
No	24
Taxane/platinum regimen	
TC	17
TEC	7
TAC	4
AP	3
DC	1
Response to taxane/platinum regimen	
Refractory	6
Resistant	22

### Chemotherapeutic response and patients’ survival

**Fig. 2:**
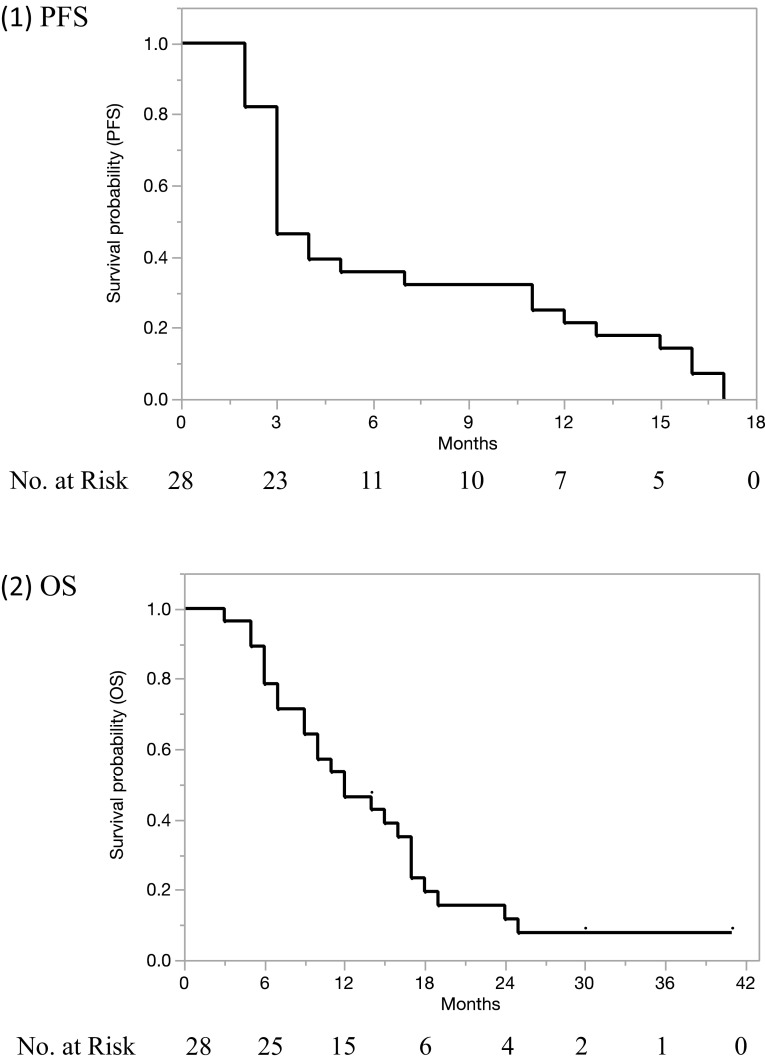
Patient survival response. The median PFS and OS were 3 months [95% confidence interval (CI) 3–7] and 12 months (95% CI 9–17), respectively. The median follow-up period was 12 months (range 3–41)

In total, 28 patients received 143 cycles of GLIF. Their treatment response, after three cycles of chemotherapy, is shown in Table 3. Response rate (CR + PR) and disease control rate (CR + PR + SD) were 7.1% (2/28) and 39.3% (11/28), respectively. As shown in Fig. 2, the median PFS and OS were 3 months (95% CI 3–7) and 12 months (95% CI 9–17), respectively. All 28 patients had relapsed by the end of this study. Twenty-four of the 28 patients died due to disease progression. Three patients were lost to follow-up, and one patient was still alive with disease. The median follow-up period was 12 months (range 3–41).

**Table 3:** Anti-tumor response (response and disease control rates)

Chemotherapeutic response	Number of patients
CR	0
PR	2
SD	9
PD	17

### Toxicities

The results of hematologic and non-hematologic toxicities are shown in Table 4. Febrile neutropenia or grade 4 neutropenia was observed in 14% (4/28). Dose adjustment due to grade 4 neutropenia was required in two patients (7.1%). Neutropenia (grade ≥ 3) occurred in 50% of the 28 patients (14/28), and grade 3 or 4 thrombocytopenia was not observed. Grade 3 anemia occurred in three patients (10.7%). Grade 3 AST/ALT was observed in one patient (3.6%). Although elevated γ-GTP (grade ≥ 3) was observed in two patients (7.1%), these events were due to disease progression. One patient demonstrated chemotherapy-induced reactivation of hepatitis B virus. No treatment-associated deaths occurred.

**Table 4:** Hematologic and non-hematologic toxicities

		*n* = 28				
**Adverse effect**		**Grade 1**	**Grade 2**	**Grade 3**	**Grade 4**	**Grade ≧ 3** (%)
Hematologic toxicities						
	Neutropenia	0	7	12	2	14 (50%)
	Febrile neutropenia	0	1	1	0	1 (3.6%)
	Anemia	11	11	3	0	3 (10.7%)
	Thrombocytopenia	2	1	0	0	0 (0%)
Non-hematologic toxicities						
	Creatinine	1	0	0	0	0 (0%)
	AST	7	1	1	0	1 (3.6%)
	ALT	9	1	1	0	1 (3.6%)
	T-Bil.	0	0	1	0	1 (3.6%)
	γ-GTP	6	3	1	1	2 (7.1%)
	ALP	6	2	0	0	0 (0%)
	Hypernatremia	0	0	0	0	0 (0%)
	Hyperkalemia	5	1	0	0	0 (0%)
	Hyponatremia	12	0	0	0	0 (0%)
	Hypokalemia	3	0	0	0	0 (0%)
	Fever	4	0	0	0	0 (0 %)
	Nausea/vomiting	18	2	0	0	0 (0%)
	Diarrhea	1	0	0	0	0 (0%)
	Peripheral neuropathy	4	0	0	0	0 (0%)
	Myalgia/arthralgia	4	0	0	0	0 (0%)
	Rash	5	0	0	0	0 (0%)
	Skin hyperpigmentation	1	0	-	-	-
	Arrhythmia	1	1	0	0	0 (0%)

## Comments

Advanced and recurrent endometrial cancers generally carry a poor prognosis. In particular, progression during treatment and shorter treatment-free intervals (TFIs) before progression are associated with poor prognosis. We were among the first to provide evidence that the majority of refractory or resistant diseases, if they progress during a first-line taxane/platinum chemotherapy (with or without anthracycline), or recur within 6 months of taxane/platinum, are non-responsive to the current regimens of second-line chemotherapy [13]. We also reported that prognosis for patients with recurrence within 6–12 months was worse relative to those relapsing at 12 months or later [14].

Although multiple-agent chemotherapy regimens that include platinum- or taxane-based chemotherapeutic agents, such as carboplatin/paclitaxel (TC), cisplatin/doxorubicin (AP), cisplatin/doxorubicin/paclitaxel (TAP), or carboplatin/docetaxel (DC) are preferred for patients with recurrent endometrial cancer [15, 16], the most appropriate regimen after failure in platinum- and/or taxane-based treatment remains unclear. We have previously demonstrated that patients with TFIs of less than 6 months after taxane/platinum-containing chemotherapy were considered to be “taxane/platinum resistant” [14]. These cases did not respond to re-administration of taxane/platinum-containing chemotherapy, and their prognosis was extremely poor.

The efficacy of AP as a second-line regimen for recurrent endometrial cancer has been investigated previously [17]. The median OS of patients treated by second-line AP, following first-line TC therapy, was 12 months. However, 40% of these patients had TFIs ≥ 6 months and were thus considered to be “partially sensitive” to platinum-based chemotherapy. Given the transient responses with platinum- or taxane-based regimens, there has been an important, but unmet need for a second-line regimen for endometrial cancer cases no longer sensitive to taxane/platinum regimens.

In the present study, a GLIF combination chemotherapy, consisting of gemcitabine, levofolinate, irinotecan, and 5-FU, was found to be both safe and effective for taxane/platinum-resistant or -refractory endometrial disease. Response and disease control rates of 7.1% tended to be better than the 0% rate for patients with a TFI of < 6 months found in our previous retrospective study [13] of variously treated patients, conducted before the introduction of GLIF therapy. The median PFS and OS after GLIF therapy were 3 months (95% CI 3–7) and 12 months (95% CI 9–17), respectively, which also tended to be longer than the 2 months (0–9 months) and 5.5 months (2–44 months), respectively, found in that previous study [13]. Moreover, febrile neutropenia or grade 4 neutropenia was demonstrated to be acceptable.

Recent phase-II studies have investigated the efficacy of molecular-targeted drugs for advanced/recurrent endometrial cancer [18, 19]. The median PFS of patients who were treated with temsirolimus was 3 months and disease control rate was 35% [18], which is similar to that of our treatment. Another phase-II trial demonstrated that the median PFS and OS of recurrent or persistent endometrial cancer patients who were treated with selumetinib were 2.3 and 8.5 months, respectively [19]. According to these recent phase-II trials, the clinical efficacy of molecular-targeted drugs remains unclear, although their toxicity seems to be tolerable.

In conclusion, we have demonstrated that GLIF combination chemotherapy was potentially beneficial and could be a treatment option for patients with advanced or recurrent endometrial cancer that is not sensitive to taxane/platinum regimens.

## Abbreviations

AP: Doxorubicin (anthracycline) plus cisplatin
CI: Confidence interval
CR: Complete response
DC: Docetaxel plus carboplatin
GLIF: Gemcitabine, levofolinate, irinotecan, and 5-fluorouracil
OS: Overall survival
PD: Progressive disease
PFS: Progression-free survival
PR: Partial response
RR: Responsive rate
SD: Stable disease
TAP: Paclitaxel, doxorubicin (anthracycline) plus cisplatin
TC: Paclitaxel plus carboplatin
TFI: Treatment-free interval
TEC: Paclitaxel, epirubicin plus carboplatin
